# Palladium (III) Fluoride Bulk and PdF_3_/Ga_2_O_3_/PdF_3_ Magnetic Tunnel Junction: Multiple Spin-Gapless Semiconducting, Perfect Spin Filtering, and High Tunnel Magnetoresistance

**DOI:** 10.3390/nano9091342

**Published:** 2019-09-19

**Authors:** Zongbin Chen, Tingzhou Li, Tie Yang, Heju Xu, Rabah Khenata, Yongchun Gao, Xiaotian Wang

**Affiliations:** 1 Department of Physics, College of Science, North China University of Science and Technology, Tangshan 063210, China; chen.12345@126.com (Z.C.); xuheju@126.com (H.X.); 2 School of Physical Science and Technology, Southwest University, Chongqing 400715, China; tzli97@163.com (T.L.); yangtie@swu.edu.cn (T.Y.); 3 Laboratoire de Physique Quantique de la Matière et de Modélisation Mathématique, Université de Mascara, Mascara 29000, Algeria; khenata_rabah@yahoo.fr

**Keywords:** spin-gapless semiconductors, magnetic tunnel junction, non-equilibrium Green’s function, first-principle calculations

## Abstract

Spin-gapless semiconductors (SGSs) with Dirac-like band crossings may exhibit massless fermions and dissipationless transport properties. In this study, by applying the density functional theory, novel multiple linear-type spin-gapless semiconducting band structures were found in a synthesized $R\overset{-}{3}c$-type bulk PdF_3_ compound, which has potential applications in ultra-fast and ultra-low power spintronic devices. The effects of spin-orbit coupling and on-site Coulomb interaction were determined for the bulk material in this study. To explore the potential applications in spintronic devices, we also performed first-principles combined with the non-equilibrium Green’s function for the PdF_3_/Ga_2_O_3_/PdF_3_ magnetic tunnel junction (MTJ). The results suggested that this MTJ exhibits perfect spin filtering and high tunnel magnetoresistance (~5.04 × 10^7^).

Spintronics [1] is a promising research field that focuses on electrons in a degree of freedom (i.e., spin). Conventional electronic devices have high power consumption and a relatively low circuit integration, resulting in physical limitations. Generally, spintronics strives for ultra-efficient and ultra-fast devices [2,3,4,5]. Thus, the development of corresponding spintronic materials is crucial and must be realized. For the past several years, diluted magnetic semiconductors [6], half-metals [7], and topological insulators [8] were proposed, and while they can each meet various requirements of spintronics, they cannot meet all of them.

In 2008, Wang [2] proposed a new spintronic material—spin-gapless semiconductors (SGSs) [9]. In general, SGSs possess a gapless feature in one spin channel and a semiconducting gap in the other spin channel. In terms of differences in the dispersion of its band structures near the Fermi level, SGSs can be divided further into parabolic or Dirac-like crossing types. Parabolic SGSs show a quadratic band dispersion for gapless bands, while Dirac-like crossing SGSs (DSGSs) exhibit a linear band dispersion at the Fermi level in one spin channel. Due to this unique dispersion, the effective electron quantity can be eliminated in DSGSs. As a member of the topological non-trivial family, DSGSs have 100% spin polarization, high Fermi velocity (*v_F_*), massless fermions around the Fermi level, and no dissipation characteristics. These desirable characteristics indicate possible applications in future high efficiency spintronic devices. 

In the past (and based on first-principle calculations), DSGSs with a single Dirac-like crossing could be observed in two-dimensional Ni_2_C_24_S_6_H_12_ [10] and Mn_2_C_6_S_12_ [11] metal–organic frameworks as well as NiCl_3_ [12], MnX_3_ (X = F, Cl, Br, and I) [13] and YN_2_ monolayers [14]. DSGSs with more than two Dirac-like crossings can be found in CrO_2_/TiO_2_ heterostructures [15] and three-dimensional MnF_3_ materials [16]. Compared with DSGSs that have a single Dirac-like crossing, DSGSs with multiple Dirac band dispersions have a stronger nonlinear electromagnetic response and a higher carrier transport efficiency at the Fermi level.

In this study, we proposed a new rhombohedral-type of bulk material, PdF_3_, which demonstrates the spin-gapless semiconducting feature with multiple linear band dispersions, by means of the density functional theory (DFT) method. We should point out that this material with $R\overset{-}{3}c$ has been prepared by the reaction of dry fluorine on PdI_2_ at about 400 °C [17]. Its magnetism and physical stability under the spin-orbit coupling effect and mechanic pressure was also investigated in detail. Additionally, we built a PdF_3/_Ga_2_O_3_/PdF_3_ magnetic tunnel junction (MTJ) and investigated its nonequilibrium spin injection and spin-polarized quantum transport properties based on the non-equilibrium Green’s function (NEGF) and in combination with first-principles calculations. The results showed that the MTJ exhibits a perfect spin-filtering feature with extremely high magnetoresistance. Details about the computational and simulation models can be found in the supplementary materials (Supplementary Figures S1 and S2).

The electronic structures of bulk PdF_3_ were calculated without the effect of spin-orbit coupling (Figure 1). We found that its electronic structure shows multiple spin-gapless semiconducting features with linear dispersion, which indicates excitation at the Fermi level owning the dissipationless and massless features. Considering that the correlation effect from the 4*d* orbit of a Pd element cannot be regarded, we also adapted DFT plus U to conduct this calculation. In this study, several Hubbard U values (from 0 to 4 eV) were tested on-site for Pd-4*d* orbits. The corresponding band structures (Supplementary Figure S3) were not significantly different from the gapless spin-up structures, and the enlargement of the gap in the spin-down channel stabilized the semiconducting feature. Therefore, we will focus on the Perdew−Burke−Ernzerhof (PBE) results in the following discussion. Also, the modified Becke–Johnson exchange potential with the generalized gradient approximation (GGA) method was also used in this work to examine the electronic structure of PdF_3_ bulk and the results are shown in Supplementary Figure S4.

As shown in Figure 1, there is clearly a semiconducting gap at about 0.92 eV in the spin-down channel, and a gapless configuration in the spin-up channel. In total, ten linear band crosses appear exactly at the Fermi level. Some of these linear crosses are located at the high symmetry points (one at the L point and another near the Z point along the Z–F1 line), and some appear along the high symmetry lines (M–G, G–Y, I–G, G–I1, F1–G, G–X1, Y|X–G, and G–N lines respectively). Also, one can see that the crossing bands have a very large linear energy range (up to 1 eV). As was the case with bulk ReO_2_ [18], hourglass-shaped band dispersions are apparent along the Z–F_1_ and G–N lines. Such an irregular distribution of band crosses and linear SGS features bring the anisotropy, but high Fermi velocities, in the whole Brillouin zone. Considering the rotation symmetry of the Brillouin zone, these linear crosses will form the Dirac rings and induce a more intensive nonlinear electromagnetic response than that induced by other materials containing a single Dirac cone. Moreover, a higher efficiency of carrier transport will exist at the Fermi level via the multiple crosses.

As shown in Figure 2, we computed the spin-resolved total density of states (TDOS) and partial density of states (PDOS) spectra. From the TDOS, a valley can be seen at the Fermi level in the spin-up channel, and the major distribution of spin-down states is below −0.7 eV and above 0.2 eV, thus forming a semiconducting gap. The spin-polarization rate [19] can be obtained by $P=\frac{\left|\rho_{↑}-\rho_{↓}\right|}{\left|\rho_{↑}+\rho_{↓}\right|}$, where $\rho_{↑/↓}$ represents the states of the spin-up or spin-down channels near the Fermi level. Here, *P* is 100%. In PDOS, the electronic structures near the Fermi level are dominated by the *p-d* hybridization between Pd and F atoms.

To calculate the effect of spin-orbit coupling, we examined the band structures (Supplementary Figure S5). As shown in this figure, the impact of SOC on these linear band crosses of PdF_3_ was very weak. Meanwhile, we considered the uniform strain effect to examine the band structural stability of PdF_3_ (Supplementary Figure S6). It was apparent that as stress is applied, some linear crossings (such as the gapless crossings along I–I_1_ under 8 GPa) are opened. In general, the characteristics of the spin-gapless behaviors in the spin-up channel still exist for PdF_3_ based on our calculated band structures.

Due to the large band gap, Ga_2_O_3_ is one of the materials that have received significant attention in recent years [20]. Further, the lattice mismatch between PdF_3_ and Ga_2_O_3_ is less than 5%, and therefore, in this work, we built a PdF_3_/Ga_2_O_3_/PdF_3_ magnetic tunnel junction (MTJ) to conduct the corresponding spin-transport calculation (Supplementary Figure S2). Figure 3 presents the calculated current–voltage curves of the MTJ in the parallel magnetization configuration (PC) and the antiparallel magnetization configuration (APC) between two electrodes. For PC, only the spin-up current contributes to the total spin-current in the range of considered bias voltage (from 0.0 to 0.1 V), and the spin-down current is strongly inhibited. In summary, the spin-up current increased rapidly when accompanied by an increase in bias voltage, while the turning decreased after the bias voltage increased up to 0.09 V. For APC, it does not matter if the spin-up and spin-down currents are both blocked.

From the results of the spin-dependent current for PC and APC, the bias-voltage-dependent spin-injection efficiency (SIE) [21] can be obtained by $SIE=\left|\frac{I^{up}-I^{down}}{I^{up}+I^{down}}\right|\times 100\%$, as shown in Figure 4a. In PC, SIE maintains 100% consistently. This can be attributed to the electronic structures of the two electrodes; the spin-down gap blocks the leap of carriers, thus making *I^down^* vanishingly small. In APC, SIE at an equilibrium state is about 10% rather than 0% due to the broken mirror symmetry of this MTJ. When the bias voltage is applied, SIE increases to nearly 90%. Moreover, the fluctuation of *I^up/down^* notably causes a decrease in SIE at the range of bias voltage from 0.4 V to 0.5 V.

In our study, a tunnel magnetoresistance (TMR) ratio [21] under finite bias is defined as $TMR=\left|\frac{I_{PC}^{tot}-I_{APC}^{tot}}{\min(I_{PC}^{tot},I_{APC}^{tot})}\right|\times 100\%$. Figure 4b exhibits the TMR ratio versus bias voltage under equilibrium and nonequilibrium states. Here, for a PdF_3_/Ga_2_O_3_/PdF_3_ MTJ, the obtained equilibrium state TMR is about 5.04 × 10^7^%, while the nonequilibrium state TMR is in the order of magnitudes of 10^11^%. Also shown in Figure 4b is the calculated TMR of about 4.11 × 10^11^% at a bias of 0.01 V. Most importantly, the high TMR in this MTJ is very stable in the range of bias voltage variation from 0.01 V to 0.1 V.

To further understand the equilibrium state of the TMR, the transmission coefficient as a function of electron energy at a finite bias voltage range is shown in Supplementary Figure S7. In PC, results reveal that the transmission coefficient of spin-up electrons at the Fermi level is much higher than that of spin-down electrons, showing that the major contribution of current comes from the spin-up electrons. In APC, the transmission coefficients of both spin-up and spin-down electrons are almost equal and vanishingly small, inducing an imperceptible current compared with that in PC. In summary, the transmission coefficient in APC is much smaller than that in PC, so an extremely high TMR can be obtained.

Furthermore, we calculated the nonequilibrium transmission spectrum T(E,V). It is worth noting that the aforementioned spin-polarized current is obtained according to integral transmission coefficients at the bias voltage window of −eV/2≤E≤eV/2 which is to say, I~∫T(E,V)dE Supplementary Figure S8 shows the nonequilibrium transmission spectrum under the bias voltage value of 20 mV, 40 mV, 60 mV, and 80 mV, respectively. One can see that the PC transmission curves within the bias window have not changed much. Also, the APC is still much lower than the PC within the bias window; therefore, an ultra-high TMR ratio can be maintained under a broad range of bias. Meanwhile, due to the enhancement of the broken geometric symmetry of this MTJ, the increased bias voltage will intensify the division of transmission curves in APC. Moreover, there is an obvious decrease in the spin-up curve in APC within the bias voltage window, while the spin-down curve is not affected. This induces a rapid increase in spin-injection efficiency, with bias voltage increasing.

Finally, as shown in Figure 5, the local density of states (LDOS) of this MTJ was calculated and summed up along the *z*-axis. The insulating gap of Ga_2_O_3_ results in a central barrier without density of states. Figure 5a demonstrates that in PC, the spin-up direction is free, and several spin-up electrons are able to tunnel in the central scattering zone from the left electrode and into the right electrode, indicating the formation of a transport channel. Therefore, the spin-up channel is in a majority spin state. As shown in Figure 5b, there are few densities of states in the two electrodes in the spin-down channel. In other words, the spin-down channel is suppressed (minority spin state), and the spin-down electrons are difficult to transmit via the magnetic electrode by tunneling through the Ga_2_O_3_ barrier.

Figure 5c shows that in the APC spin-up channel, the density of states is mainly localized in the left electrode, while almost no density of states can be found in the right electrode. An explanation for this is that although the left electrode can generate spin-up electrons, there are few states in the right electrode that can accommodate these electrons; therefore, spin electrons cannot flow into the right electrode. On the contrary, in the APC spin-down channel (Figure 5d), the density of states is concentrated on the right electrode, while there is little density of states in the left electrode. This shows that although the right electrode can accept a large number of electrons, the left electrode does not provide many electrons in the spin-down channel. Therefore, when this MTJ is in the APC state, both the spin-up and spin-down channels are blocked and the spin-polarized current cannot be detected; this resulted in a high resistance state.

To summarize, we theoretically proposed a new spintronic material, rhombohedral-type PdF_3_, which has fully spin-polarized and multiple linear spin-gapless semiconducting features. The computed results reveal the robustness of its innovative electronic structures and a long spin-coherence length. These excellent features show great promise for future experimental study and realistic application. We further built the PdF_3_/Ga_2_O_3_/PdF_3_ MTJ to deeply investigate the spin-transport feature by first-principles combined with the nonequilibrium Green’s function method. The ultrahigh equilibrium and nonequilibrium states of TMR are obtained at about 5.04 × 10^7^% and 4.11 × 10^11^% (at 0.01 V), respectively. Additionally, the calculation of spin-injection efficiency exhibited the perfect spin-filtering performance of this MTJ. We also utilized the current–voltage curve, transmission spectrum, and local density of states to gain a deep understanding of the physical mechanism of its spin-transport properties. In conclusion, such an MTJ is a promising candidate for realistic spintronic applications. We hope this work can provide guidance for future experimental studies.

## Figures and Tables

**Figure 1:**
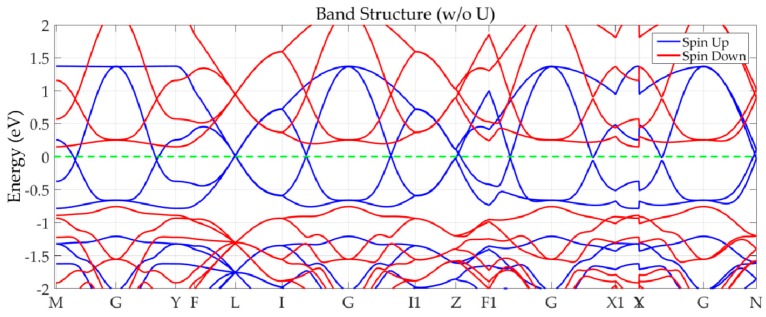
Band structure of bulk PdF_3_ calculated based on the Perdew−Burke−Ernzerhof function without consideration to the spin-orbit coupling effect.

**Figure 2:**
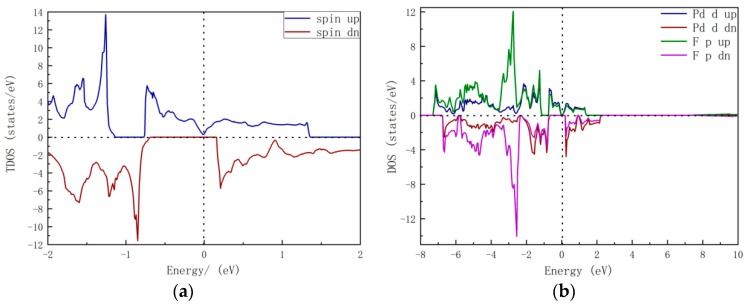
(**a**) Total and (**b**) projected density of states of rhombohedral-type PdF_3_ bulk. The Fermi level was set to zero. TDOS = total density of states.

**Figure 3:**
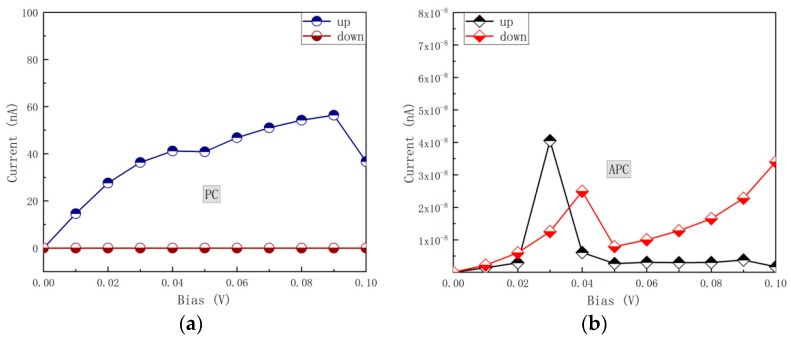
(**a**): Spin-dependent current in parallel configuration (PC) as a function of bias voltage for a PdF_3_ based magnetic tunnel junction (MTJ). (**b**): The spin-dependent current in anti-parallel configuration (APC) as a function of bias voltage for a PdF_3_ based MTJ.

**Figure 4:**
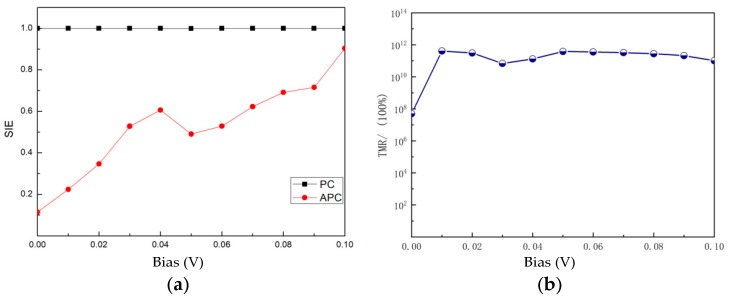
(**a**) Spin-injection efficiency (SIE) and (**b**) tunnel magnetoresistance (TMR) as a function of bias voltage for a PdF_3_ based MTJ.

**Figure 5:**
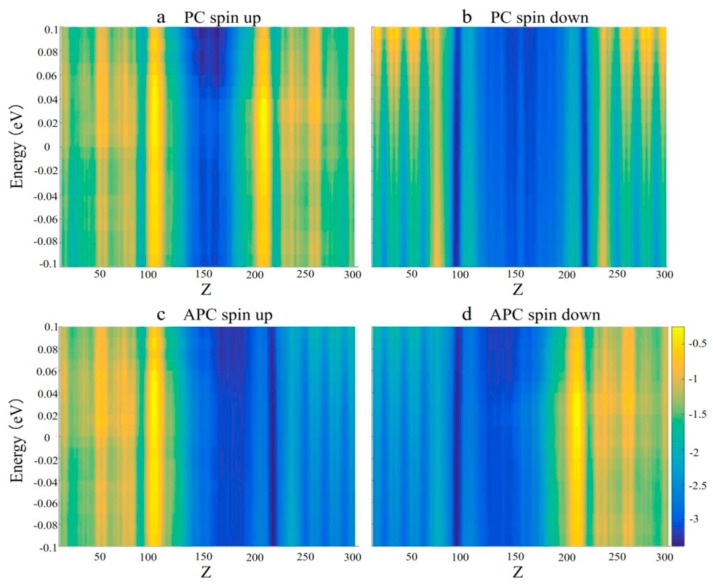
Local density of states (LDOS) for (**a**) PC spin-up; (**b**) PC spin-down; (**c**) APC spin-up; and (**d**) APC spin-down configurations for a PdF_3_ based MTJ.

## Supplementary Materials

The following are available online at https://www.mdpi.com/2079-4991/9/9/1342/s1, Figure S1: (a) Front and (b) side views of lattice structure of rhombohedral-type PdF_3_ bulk, Figure S2: Geometric structure of PdF_3_-Ga_2_O_3_-PdF_3_ magnetic tunnel junction (MTJ), Figure S3: Band structures of PdF_3_ bulk calculated based on the PBE+U method. Here, we assessed the influence brought by Hubbard U from 1 eV to 4 eV for Pd-4*d* orbits, Figure S4: Band structure of PdF_3_ bulk under MBJGGA calculation within VASP software, Figure S5: Band structure of PdF_3_ bulk calculated based on the PBE functional with consideration of the SOC effect, Figure S6: Band structures of PdF_3_ bulk under different pressures. These results were calculated based on the PBE functional, Figure S7: Equilibrium-state transmission spectrum in parallel configuration for PdF_3_-based MTJ. (b) Equilibrium-state transmission spectrum in anti-parallel configuration for PdF_3_-based MTJ, Figure S8: Non-equilibrium transmission spectrum versus electron energy at a fixed bias voltage for PdF_3_-based MTJ.
