# Supplementary material for: Palladium (III) Fluoride Bulk and PdF3/Ga2O3/PdF3 Magnetic Tunnel Junction: Multiple Spin-Gapless Semiconducting, Perfect Spin Filtering, and High Tunnel Magnetoresistance
Source: Nanomaterials (Basel). 2019 Sep 19;9(9):1342. doi: 10.3390/nano9091342 (PMC6781031; doi:10.3390/nano9091342)
Supplement: Supplementary file 1 [file nanomaterials-09-01342-s001.pdf]

Supplementary Materials

Palladium (III) Fluoride Bulk and $PdF_3/Ga_2O_3/PdF_3$ Magnetic Tunnel Junction: Multiple Spin-Gapless Semiconducting, Perfect Spin Filtering, and High Tunnel Magnetoresistance

Computational Methods

In our study, the structural, electronic, and magnetic properties for rhombohedral-type PdF_3 bulk were calculated based on the density functional theory (DFT), as implemented in the Nanodcal package [1]. The Perdew-Burke-Ernzerhof (PBE) exchange-correlation functional was applied [2] for the parameterization of general gradient approximation (GGA) [3]. The DFT+U function was considered to examine the electronic structures of PdF_3.

The spin-transport calculations were performed by the nanodcal package, which used the DFT combined with the non-equilibrium Green's Function method [4]. In this calculation, the Monkhorst-Pack k-meshes for the electrons and the centers were 9×9×100 and 9×9×1, respectively. The self-consistent calculation is limited to 10^{-5} Hartree tolerance. All these parameters were evaluated and found to be sufficient to obtain accurate results. The spin-dependent current is calculated by the Landauer-Buttiker formula:

$$I^{\uparrow(\downarrow)} = \frac{e}{h}\int_{-\infty}^{\infty} \left\{T^{\uparrow(\downarrow)}(E,V_b)\left[f_L(E-\mu_L) - f_R(E-\mu_R)\right]\right\} dE,$$

where $f_{L/R}(E-\mu_{L/R})$ is the Fermi-Dirac distribution for the left (right) electrode, and $\mu_{L/R} = E_F \pm eV/2$ is the corresponding electrochemical potential. $T^{\uparrow(\downarrow)}(E,V_b)$ is the spin-dependent transmission coefficient:

$$T^{\uparrow(\downarrow)}(E,V_b) = Tr\left[\Gamma_L G^R \Gamma_R G^A\right]^{\uparrow(\downarrow)},$$

where $G^{R(A)}$ is the retarded (advanced) Green's function of the central region, and $\Gamma_{L(R)}$ is the coupling matrix of the left (right) electrode.

Models of bulk Palladium (III) fluoride and $PdF_3/Ga_2O_3/PdF_3$ MTJ

The crystal structure of the rhombohedral-type PdF_3 (space group: $R\overline{3}c$, No. 167, ICSD ID: 16675), as shown in Figure S1. The crystal structure has been fully relaxed and detailed structure optimization methods can be found in The Materials Project database [5]. The obtained lattice constants are a = b = c = 5.618 Å, $\alpha = \beta = \gamma$ = 54.956°, respectively. The lattice parameters obtained by theory are consistent with the experimental values [6]. According to the database [5], one can see that the calculated formation energy is -1.592 eV and negative value of formation energy shows the possible stability of bulk Palladium (III) fluoride. The magnetic ground state of PdF_3 is ferromagnetic with a magnetic moment of 0.779 μ_B.

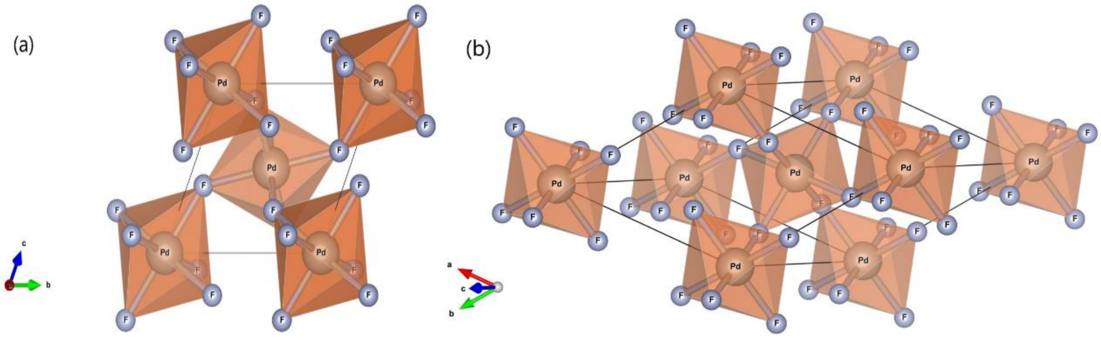

Figure S1. (**a**) Front and (**b**) side views of lattice structure of rhombohedral-type PdF_3 bulk.

In our work, the MTJ device model is periodic along the x- and y-axes, while the transport direction is along the *z*-axis. The crystals of Ga_2O_3 can be found here [7]. Ga_2O_3 with rhombohedral-type is a semiconductor and no virtual frequency in the phonon spectrum of Ga_2O_3 guarantee the stability of this material. The optimized distance between PdF_3 layer with F-terminated interface and O layer was found to be 2.61 Å. The lattice mismatch between PdF_3 and Ga_2O_3 is 3.72%.

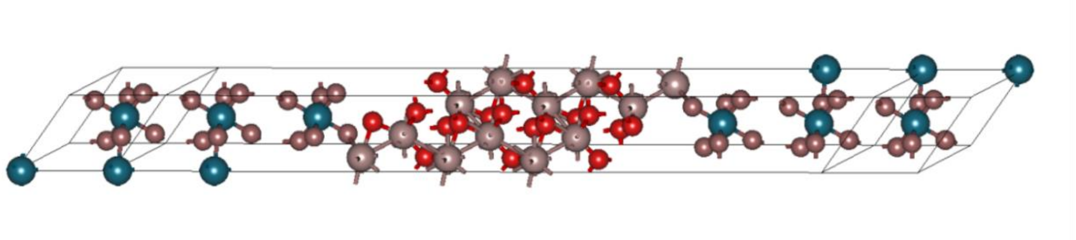

Figure S2. Geometric structure of PdF_3-Ga_2O_3-PdF_3 magnetic tunnel junction (MTJ).

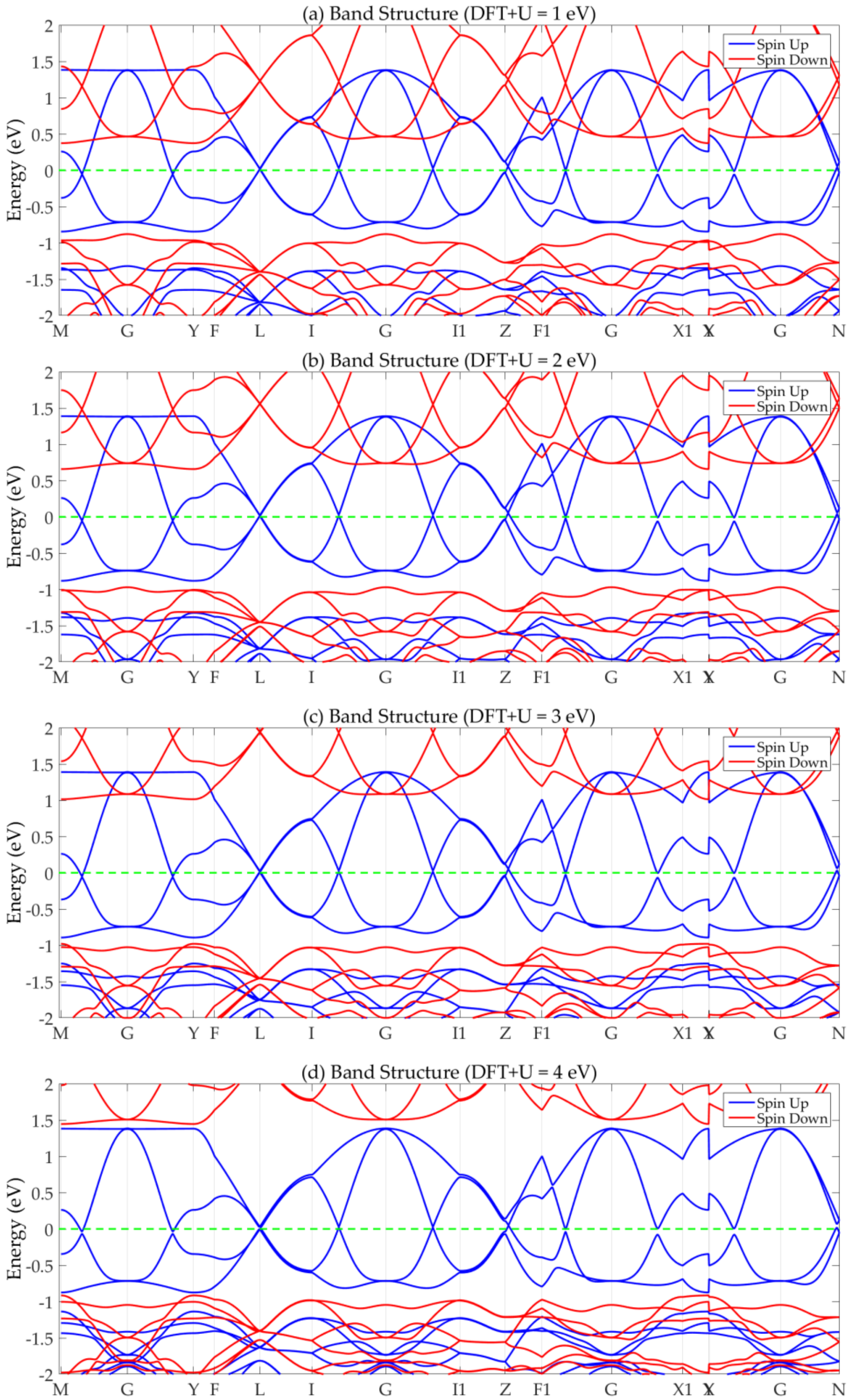

Figure S3. Band structures of PdF_3 bulk calculated based on the PBE+U method. Here, we assessed the influence brought by Hubbard U from 1 eV to 4 eV for Pd-4*d* orbits.

Band structure under MBJ potential

Since the GGA potential sometimes underestimates potential band gaps, here we check the band structure of PdF_3 by using a combination of modified Becke-Johnson exchange potential with GGA (MBJGGA) [8]. As shown in Figure S4, we can observe that the novel multiple linear-type spin-gapless semiconducting band structures in PdF_3 retain under the MBJ calculation.

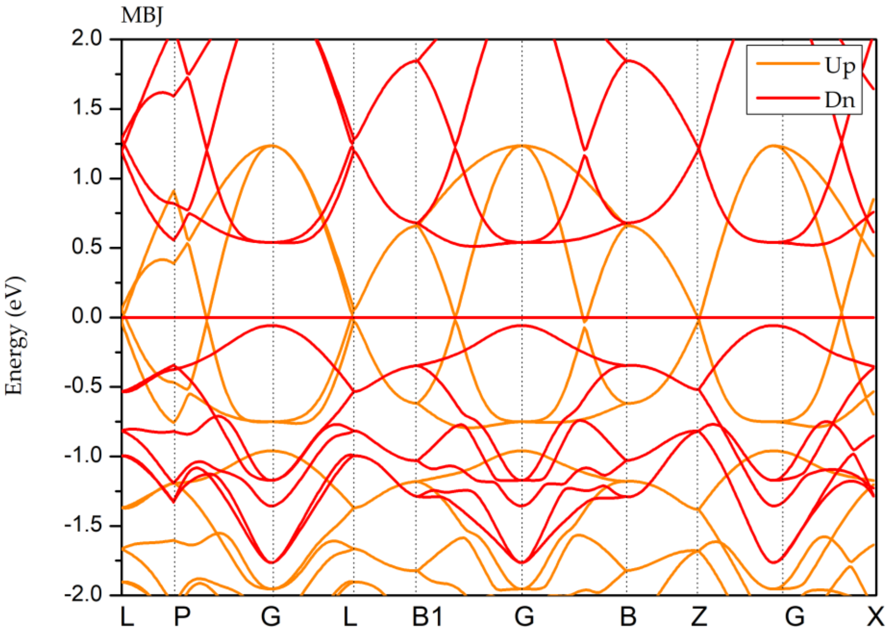

Figure S4. Band structure of PdF_3 bulk under MBJGGA calculation within VASP software.

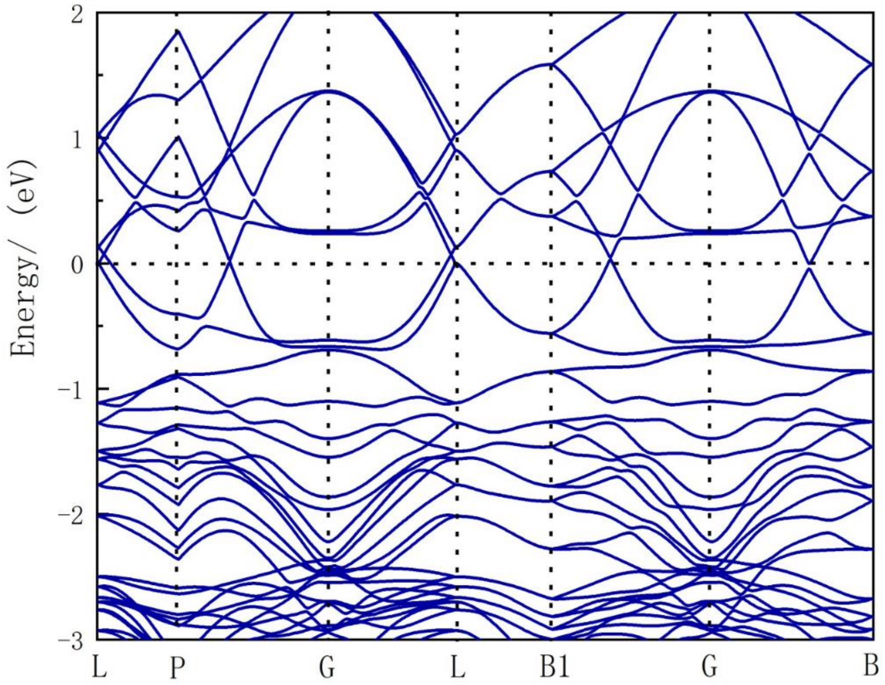

Figure S5. Band structure of PdF_3 bulk calculated based on the PBE functional with consideration of the SOC effect.

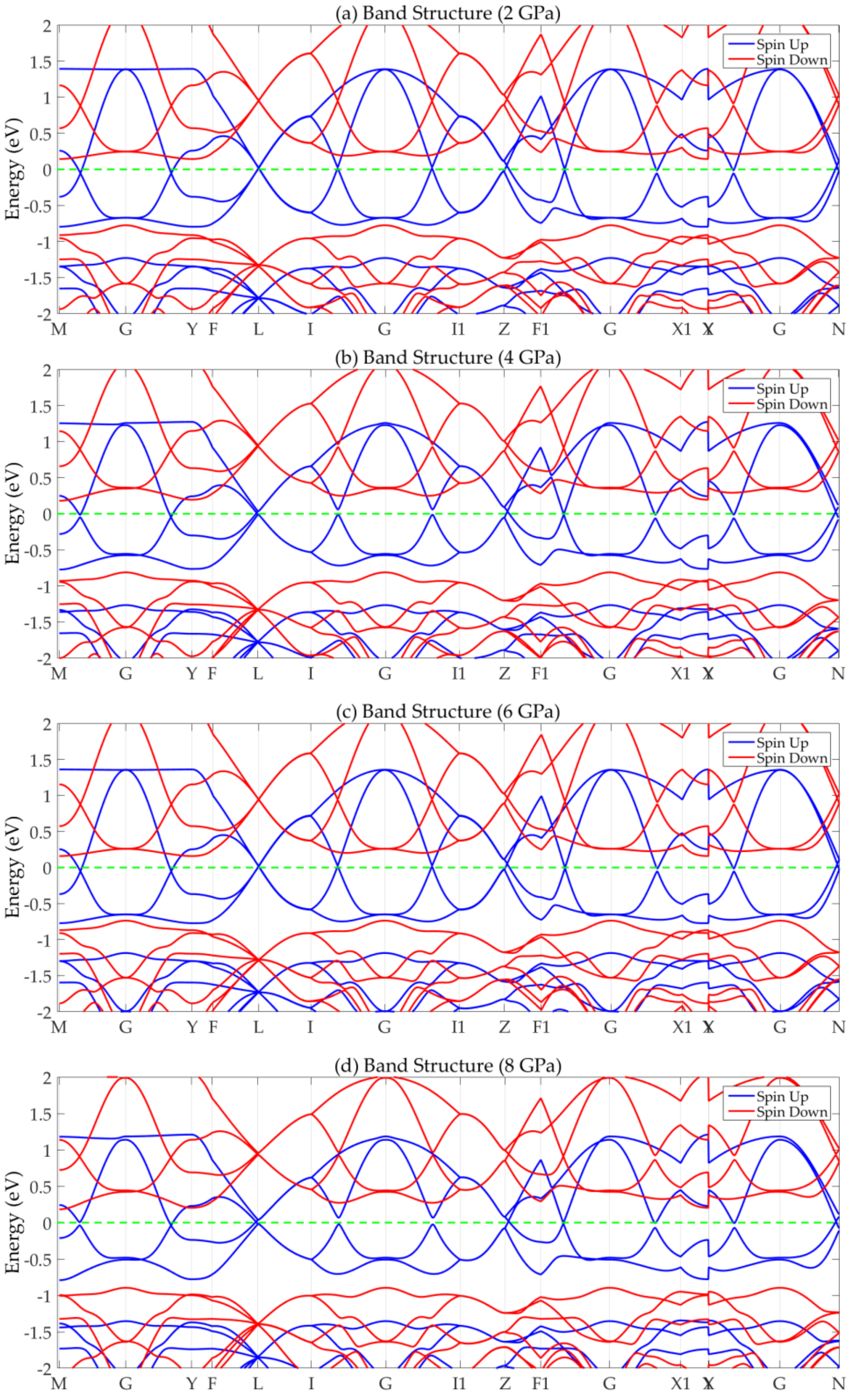

Figure S6. Band structures of PdF_3 bulk under different pressures. These results were calculated based on the PBE functional.

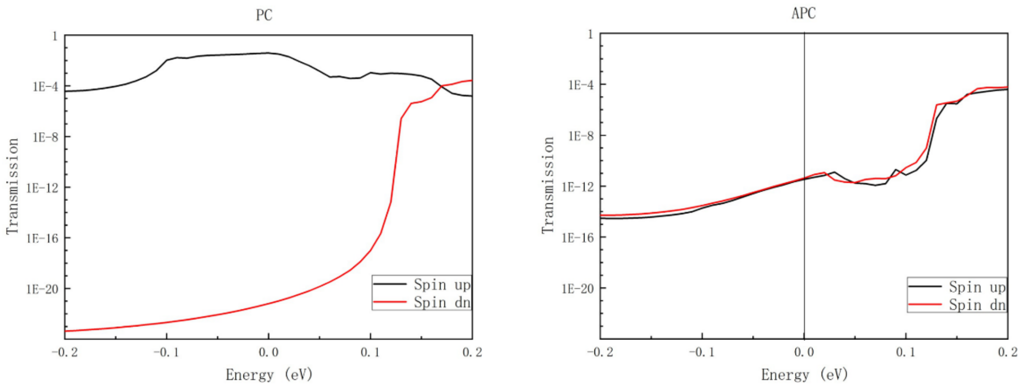

(**a**) (**b**)

Figure S7. (**a**) Equilibrium-state transmission spectrum in parallel configuration for PdF_3-based MTJ. (**b**) Equilibrium-state transmission spectrum in anti-parallel configuration for PdF_3-based MTJ.

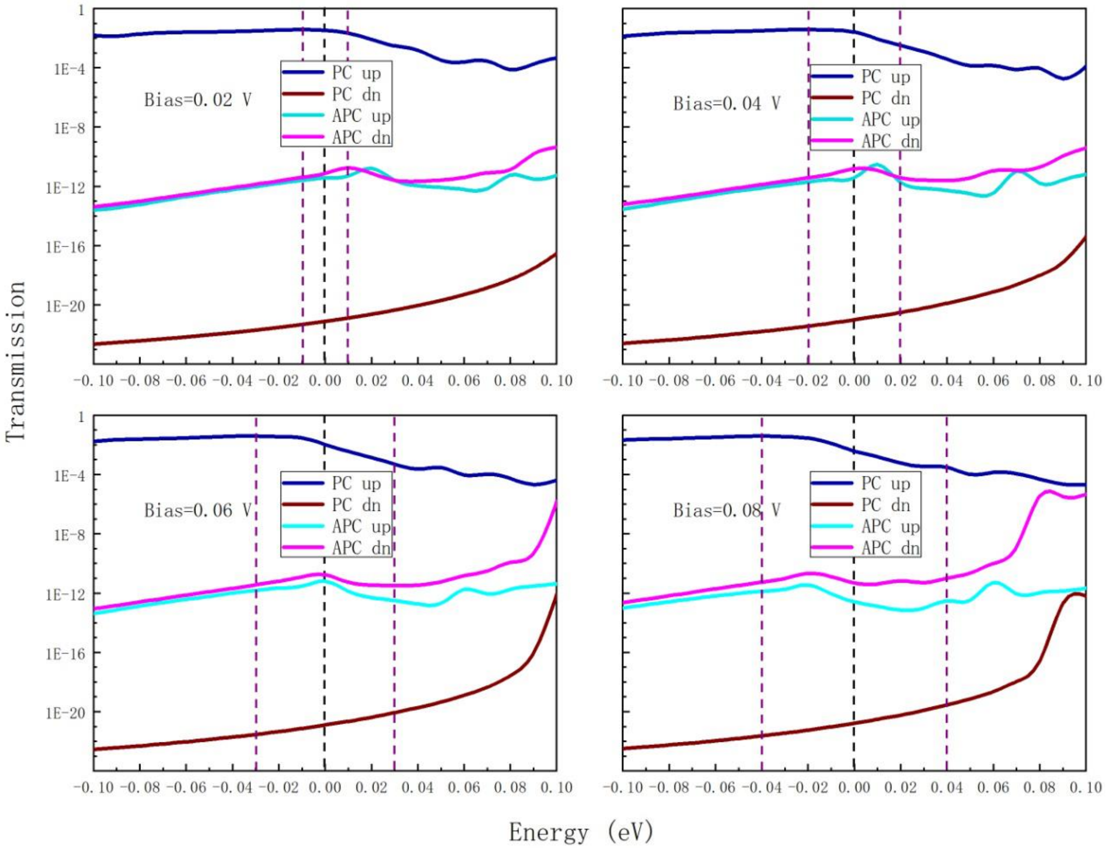

Figure S8. Non-equilibrium transmission spectrum versus electron energy at a fixed bias voltage for PdF_3-based MTJ.

References

1. Taylor, J.; Guo, H.; Wang, J. Ab initio modeling of quantum transport properties of molecular electronic devices. *Phys. Rev. B* **2001**, *63*(24), 245407.
2. Perdew, J. P.; Burke, K.; Ernzerhof, M. Perdew, burke, and ernzerhof reply. *Phys. Rev. Lett.* **1998**, *80*, 891.
3. Perdew, J. P.; Burke, K.; Ernzerhof, M. Generalized gradient approximation made simple. *Phys. Rev. Lett.* **1996**, *77*, 3865.
4. Brandbyge, M.; Mozos, J. L.; Ordejón, P.; Taylor, J.; Stokbro, K. Density-functional method for nonequilibrium electron transport. *Phys. Rev. B* **2002**, *65*(16), 165401.
5. https://www.materialsproject.org/materials/mp-13679/
6. http://www.crystallography.net/cod/2310600.html
7. https://www.materialsproject.org/materials/mp-1243/
8. Traoré, B.; Bouder, G.; Lafargue-Dit-Hauret, W.; Rocquefelte, X.; Katan, C.; Tran, F.; Kepenekian, M. Efficient and accurate calculation of band gaps of halide perovskites with the Tran-Blaha modified Becke-Johnson potential. *Phys. Rev. B* **2019**, *99*, 035139.
